# Making Sense in Antisense: Therapeutic Potential of Noncoding RNAs in Diabetes-Induced Vascular Dysfunction

**DOI:** 10.1155/2013/834727

**Published:** 2013-11-27

**Authors:** Suzanne M. Eken, Hong Jin, Ekaterina Chernogubova, Lars Maegdefessel

**Affiliations:** Atherosclerosis Research Unit, Department of Medicine, Center for Molecular Medicine (CMM L8), Karolinska Institute, 17176 Stockholm, Sweden

## Abstract

The rapid rise of type II diabetes mellitus and its accompanying vascular complications call for novel approaches in unravelling its pathophysiological mechanisms and designing new treatment modalities. Noncoding RNAs represent a class of previously unknown molecular modulators of this disease. The most important features of diabetes-induced vascular disease, which include metabolic deregulation, increased oxidative stress, release of inflammatory mediators like adipokines, and pathologic changes in vascular cells, all are depicted and governed by a certain set of noncoding RNAs. While these mechanisms are being unravelled, new diagnostic and therapeutic opportunities to treat diabetes-induced vascular disease emerge.

## 1. Prevalence of Diabetes and Vascular Complications

The prevalence of type II diabetes mellitus (T2DM) and related metabolic syndrome keeps rising at an alarming rate and becomes a global health issue affecting children, adolescents, and adults. According to the World Health Organization, approximately 346 million people worldwide have T2DM, and this number is estimated to almost double by 2030 [1, 2]. T2DM is a progressive multisystem disease accompanied by vascular dysfunction and a tremendous increase in cardiovascular mortality [3]. Patients with diabetes and/or metabolic syndrome have a significantly increased risk of cardiovascular complications compared to people with normal insulin sensitivity and production. In the past years, many studies have tried to reveal the mechanisms for diabetic vascular complications, with varying degrees of success. The recent discovery of noncoding RNA (ncRNAs, e.g., microRNAs and long noncoding RNAs), as well as their influence on human pathophysiology, provides us with new opportunities to unravel and positively influence the disease process. In the present review, we summarize the pathophysiology of T2DM associated vascular disease and highlight the association of ncRNA with diabetic vascular dysfunction.

## 2. Pathophysiology of Diabetic Vascular Disease

The complications of T2DM encompass a diverse range of pathologies of large and small arteries, leading to diabetic macrovascular occlusive disease and/or microvascular dysfunction, which include coronary artery diseases, cerebral artery diseases, and peripheral vascular diseases amongst others [1, 4]. Until now, most common additive risk factors for vascular disease in people with diabetes have been demonstrated as hyperglycaemia, insulin resistance, dyslipidaemia, hypertension, tobacco use, and obesity [2, 5]; however, the interaction of the factors and molecular signalling pathways have not been fully elucidated. Established mechanisms for vascular disease in diabetic patients are manifold and include the pathologic effects of advanced glycosylation end product (AGE) accumulation, impaired vasodilator response attributable to nitric oxide inhibition, smooth muscle cell dysfunction, overproduction of endothelial growth factors, chronic inflammation, hemodynamic deregulation, impaired fibrinolytic ability, and enhanced platelet aggregation [5].

### 2.1. Insulin Signalling and Hyperglycaemia

Abnormalities in vascular endothelial (EC) and vascular smooth muscle cell (VSMC) function, as well as a propensity to thrombosis, are important contributors to vascular complications [5]. Hyperglycaemia and insulin resistance have been identified as key players in the development of diabetic atherosclerosis, with metabolic insulin signalling being an important contributor to normal vascular function and homeostasis [5]. Decreased insulin sensitivity in cardiovascular tissues is an underlying abnormality in obesity, hypertension, and T2DM [3]. In physiological conditions, insulin promotes endothelium-dependent relaxation, by a mechanism that involves an increase of nitric oxide (NO) production via activation of phosphatidylinositol-3 kinase (PI3K) and Akt kinase pathways [6, 7]. NO has also been shown to prevent endothelial apoptosis, as well as neutrophil and platelet adhesion to the vascular wall [8]. The initial trigger, by which high glucose concentrations alter vascular function, is the imbalance between nitric oxide (NO) bioavailability and accumulation of reactive oxygen species (ROS) [9]. Decrease in NO bioavailability is considered the hallmark of endothelial dysfunction, subsequently leading to attenuated vascular relaxation and atherosclerosis [10].

Insulin signalling also plays a critical role in normal vascular function via modulation of calcium handling and sensitivity in VSMCs [3]. When insulin signal transduction is impaired, bioavailability of NO in ECs decreases, while endothelin-1 production, the inflammatory activity, and smooth muscle cell proliferation increase [6].

### 2.2. Dyslipidaemia

High circulating levels of triglyceride-rich particles, reduced synthesis of HDL, and enhanced production of atherogenic low-density lipoprotein (LDL) particles characterize diabetic dyslipidaemia [11, 12]. However, the regulation of lipid metabolism in diabetes is extremely complex and the mechanisms to trigger vascular dysfunction are only partially explored. The fading of the glycocalyx in large arteries exposed to hyperlipidaemic stress may be an early characteristic of an increased vascular vulnerability [13]. Also, high levels of LDL and free fatty acids (FFAs) can cause increased permeability of ECs, induce EC-derived foam cells formation and abnormal hyperplasia of extracellular matrix, as well as disturbed secretion of NO and proinflammatory molecules, such as vascular cell adhesion molecule 1 (VCAM-1), platelet/endothelial cell adhesion molecule 1 (PECAM-1), intercellular adhesion molecule 1 (ICAM-1), P-selectin, monocyte chemoattractant protein 1 (MCP-1), interleukin 6 (IL-6), Toll-like receptor 4 (TLR-4), CD40, PAI-1, and so forth [13]. Although many clinical trials have demonstrated significant advantages of utilizing cholesterol-targeting drugs to reduce cardiovascular complications of diabetic patients [14–16], the remaining higher prevalence of vascular dysfunction calls for further basic and clinical studies, allowing for better prevention and treatment.

### 2.3. Vascular Oxidative Stress

T2DM resulting in vascular dysfunction may also occur through the increase in NAPDH oxidase-induced ROS production in the vasculature [17]. In T2DM, high intracellular glucose levels increase ROS production by triggering various cellular mechanisms and regulators, such as protein kinase C (PKC) activation, polyol and hexosamine flux, AGEs, and nuclear factor kappa B (NF-*κ*B) mediated vascular inflammation [9, 10]. Hyperglycaemia-mediated superoxide formation contributes to the pathophysiological complications in diabetic patients. Not only is the generation of reactive oxygen species (ROS) elevated in diabetes, but the activity of the antioxidant defence system also declines [18]. There are multiple targets of oxidative damage in the diabetic vasculature, with modifications of proteins, lipids, and nucleic acids occurring in both ECs and VSMCs. Enhanced oxygen radical production through tumour necrosis factor *α* (TNF-*α*) and AGE or AGE receptor (RAGE) signalling reduces NO bioavailability and results in impairment of vascular function [19]. Furthermore, via scavenger receptor recognition, ROS trigger recruitment of monocytes and their differentiation into macrophages, which initiate a vascular inflammation cascade [20].

### 2.4. Adipokines

Obesity and T2DM are associated with adverse expression patterns of various adipose-derived cytokines and chemokines and enhanced adipose inflammatory cell infiltration [21]. Adipokines produced by adipose tissue may affect vascular function and insulin sensitivity [22]. To date, several adipokines have been identified and characterized to modify vascular function, and the list is still growing [23].

Adiponectin for example, an anti-inflammatory adipokine, which is reduced in obesity and T2DM [24], has been shown to increase insulin sensitivity and to improve vascular function by reducing TNF-*α*-stimulated expression of endothelial adhesion molecules and monocyte attachment [25–27]. In addition, adiponectin attenuates the production of ROS induced by high glucose, oxidized LDL, and palmitate in endothelial cells [27].

Another well studied adipokine, leptin, has been suggested to induce vascular endothelial dysfunction [28] and VSMC proliferation [29]. Also, resistin, which is elevated in obesity and T2DM, was found to be a strong risk factor for acute coronary syndrome in different clinical studies [30]. Resistin promotes atherogenic changes in VSMCs such as increased proliferation, migration, and contractility [31]. Many other adipokines and cytokines, such as visfatin, TNF-*α*, plasminogen activator inhibitor 1 (PAI-1), and so on, may all contribute to vascular dysfunction by affecting vascular tone and infiltration of inflammatory cells [31, 32].

## 3. Noncoding RNAs

In the last two decades, our view of genetic biological regulation has changed dramatically. Transcriptomic analyses revealed that only a surprisingly minor portion of 1-2% of the human genome is protein-coding transcripts and the remaining 98% is largely transcribed into noncoding RNA, which has been identified as an immensely complex and important regulatory machinery. Among the different noncoding RNAs that exist, microRNAs (miRNAs) and long noncoding RNAs (lncRNAs) currently receive most interest, because their regulating capabilities have been shown to play a significant role in human disease [33].

miRNAs are 20- to 22-nucleotides long RNA molecules, initially discovered in the nematode worm *C. elegans* as posttranscriptional negative regulators of gene expression via antisense RNA-RNA interaction [34, 35]. Up to 60% of all mammalian genes are reported to be under miRNA influence [36]. One miRNA is capable of targeting a collection of messenger RNA (mRNA) molecules, and therefore a set of targets, for example, in a stress cascade, can be entirely miRNA governed. Disease states are typical examples of miRNA deregulation [37]. Together, these facts imply a disease modifying potential for miRNA mimics (premiRs) and inhibitors (antagomiRs), and indeed antagomiRs have already proven their therapeutic potential in preclinical and clinical trials [38, 39]. miRNAs size allows them to leave the nucleus and exert their actions at a distance from where they were transcribed. Other than mRNA that—upon entering the circulation—is prone to degradation, miRNAs associate with diverse types of carriers such as microparticles and exosomes, which allow them to be detected peripherally [40]. As a result, in addition to their therapeutic potential, a lot of research has shifted towards prospective utilization as disease biomarkers in plasma and other body fluids.

The functional roles of long noncoding RNAs (lncRNAs) are less well known and span a wider range of regulatory mechanisms. lncRNA release is triggered by a number of cellular responses. Transcription and processing of lncRNAs are similar to those of miRNAs. lncRNAs influence the regulation of gene expression by driving the formation of ribonucleic-protein complexes [41, 42]. To explain these functions, several models have been proposed [41], but the exact mechanisms have yet to be unravelled. Recent findings show that lncRNAs can function as host transcripts for miRNAs [43].

In this review we aim at summarizing recent advances in the role of miRNAs and lncRNAs in diabetes-related vascular dysfunction.

## 4. Human Arrays Investigating miRNA Associations with Diabetes

In 2010, a human plasma microarray was the first to identify miRNAs associated with incident or manifest T2DM. miRNAs that were differentially regulated between T2DM cases and controls were miR-15a, -20b, -21, -24, -28-3p, -29b, -126, -150, -191, -197, -223, -320, and -486. In particularly a loss of endothelial miR-126 was characteristic for the T2DM signature, as confirmed by the exposure of human umbilical vein endothelial cells (HUVECs) to high glucose, which led to a drop in the cells' release of miR-126 [44]. In addition, when looking specifically for miR-146a because of its relation with heme oxygenase-1 (HO-1) expression, Rong et al. recently showed that this miRNA was also significantly increased in the circulation of patients with newly diagnosed T2DM as compared to age- and sex-matched controls [45]. These different findings illustrate the importance of mechanistic studies in miRNA research to reveal the biochemical background of differences in miRNA expression. With this knowledge lacking, it is not likely that there will soon be a clinically available miRNA signature of vascular risk in T2DM.

**Figure 1:**
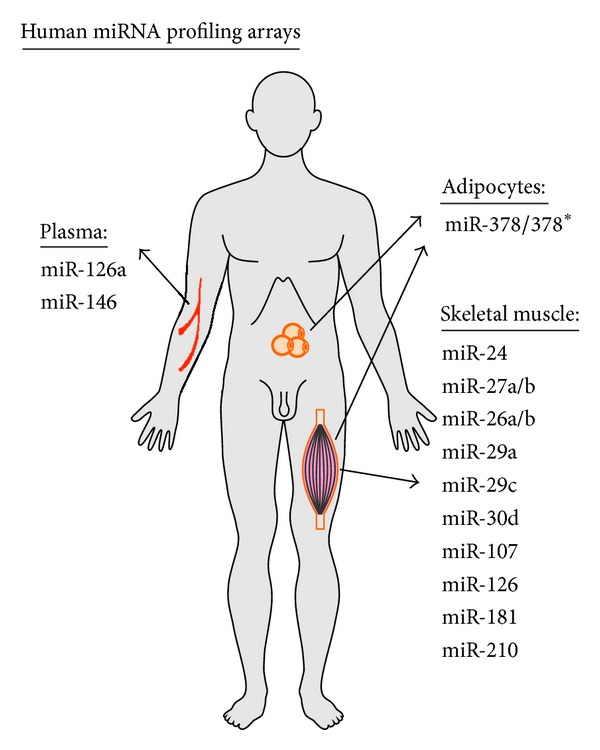
The most well-established miRNAs identified by human profiling arrays in diabetic disease.

A genome-wide association study (GWAS) of human skeletal muscle from subjects with and without T2DM revealed different expression levels of miR-15a, -15b, -98, -99a, -100a, -106b, -133a, -133b, -143, -152, -185, and -190 [46]. Insulin administration in human skeletal muscle was demonstrated causal for the decreased expression of 39 miRNAs [47]. The targets of these miRNAs are associated with insulin signalling and ubiquitination-mediated proteolysis. T2DM was shown to be associated with impaired regulation of miRNAs by insulin. Among these 39 miRNAs, miR-24, -26a, -26b, -27a, -27b, -29a, -29c, -30d, -107, -126, -181, and -210 indeed do affect vascular and diabetic parameters in *in vitro* and *in vivo* disease models, as will be discussed in the following sections.  Figure 1 depicts the most well-established miRNAs identified by human profiling arrays in diabetes.

## 5. miRNA Involvement in Metabolic Pathophysiology

**Figure 2:**
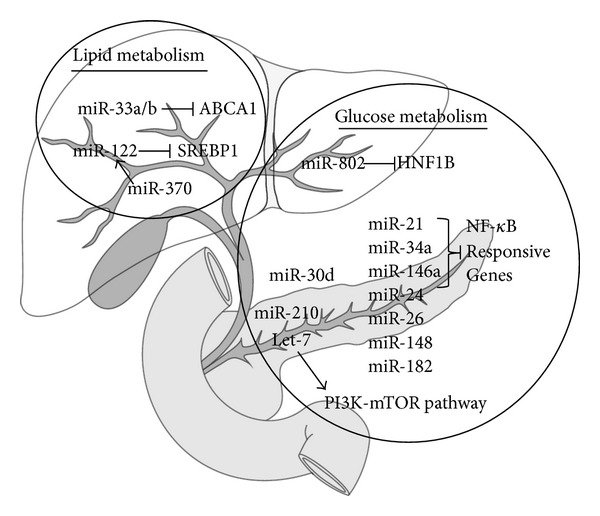
miRNAs involved in glucose and lipid homeostasis. ABCA1: a subfamily ATP-binding cassette 1; SREBP1: sterol regulatory element-binding protein 1; HNF1B: hepatocyte nuclear factor 1 *β*; NF-*κ*B: nuclear factor kappa B; PI3K-mTOR: phosphoinositide 3-kinase-mTOR.

A recent comprehensive description of miRNAs in human metabolism captured the majority of diabetes-associated miRNAs [48], and not surprisingly many of them overlap with those influencing endothelial dysfunction-related atherosclerosis. We will discuss the most-established as well as the most recently discovered miRNAs. A summary is provided in Figure 2.

### 5.1. Glucose Metabolism

The Let-7 miRNA family is a group of miRNAs acting as tumour suppressors, inhibiting a set of oncogenes and cell cycle regulators [49]. Let-7 function is inhibited by the RNA-binding proteins *Lin28a *and *Lin28b*, a regulatory capacity associated with developmental progression in nematodes [50], and also Let-7 has been shown to be crucial for physiologic glucose homeostasis, glucose tolerance, and insulin signalling by inhibiting a variety of targets in the phosphoinositide 3-kinase-mTOR (PI3K-mTOR) pathway in mouse models of obesity and T2DM [51, 52].

One of the miRNAs identified by human skeletal muscle GWAS as being associated with type II diabetes [46], miR-106b, has been further investigated regarding its role in mitofusin-2 (MFN2) mediated mitochondrial dysfunction. In a series of *in vitro* gain-of-function and loss-of-function studies in mouse C2C12 myoblasts, it was shown that by inhibiting *Mfn2*, miR-106b negatively affected mitochondrial morphology and function and increased ROS production [53].

miR-802 is upregulated in liver tissue of obese human individuals and has been shown to negatively regulate the gene encoding hepatocyte nuclear factor 1*β* (*Hnf1b*) in mice [54]. HNF1B is causally linked with maturity onset of diabetes in the young (MODY) type 5 and loss of function of this gene activates pathways involved in gluconeogenesis, *β*-oxidation of fatty acids, oxidative phosphorylation, and the tricarbonic acid cycle. By affecting HNF1B function, miR-802 counteracted glucose tolerance and insulin sensitivity, as shown *in vitro* as well as *in vivo* [54].

miR-21, -24, -126, and -146a are significant modulators of glucose metabolism in different *in vitro* and *in vivo* models of diabetes. By targeting NF-*κ*B responsive genes, miR-21, -34a, and -146a regulate cytokine-mediated *β*-cell dysfunction during the initial phases of type I diabetes in nonobese diabetic mice [55]. In mouse pancreas, miR-24, -26, -182, and -148 inhibit insulin biosynthesis via SRY-box 6 (*Sox6*) and e22 basic helix-loop-helix transcription factor (*Bhlhe22*), transcriptional repressors of insulin production [56].

In mouse insulinoma (MIN6) cells stimulated with glucose, miR-30d enhances insulin gene transcription, indicating that miR-30d could be responsible for downregulating insulin transcription repressors [57]. miR-34a, -132, -184, -199a-3p, -203, -210, -338-3p, and -383 deregulation has been shown to induce *β*-cell apoptosis in MIN6 cells, dispersed rat islet cells, and dissociated human pancreatic island cells [58, 59].

### 5.2. Lipid Metabolism

miR-33a/b and miR-122 are liver-specific miRNAs directly regulating lipid metabolism. The miR-33a and -33b sequences are hosted by the sterol regulatory element-binding protein (*SREBP*) genes 1 and 2. They negatively regulate high-density lipoprotein (HDL) cholesterol synthesis and reverse cholesterol transport viainhibition of the A subfamily ATP-binding cassette (*ABCA1*) in human liver cells. In a preclinical trial, miR-33a/b antagonism successfully lowered plasma triglycerides in non-human primates [38]. In human liver, miR-122 is the most abundantly expressed miRNA and it has important liver-specific functions that can be modulated *in vivo* with antagomiRs [39, 60]. miR-122 affects fatty acid synthesis and oxidation as well as triglyceride synthesis via AMP-activated *α*1 catalytic subunit protein kinase (*Prkaa1*), *Srebp1*, and diacylglycerol O-acyltransferase 2 (*Dgat2*) in mouse hepatic cells [61]. miR-370 increases miR-122 expression in HepG2 cells [62]. miR-17-5p, -99a, -132, -134, -145, 181a, and -197 are associated with adipose tissue morphology and key metabolic parameters in human overweight and obese individuals [63]. miR-122, a miRNA that is essential for hepatitis C virus (HCV) stability and propagation in the liver, has proven to be an effective target in HCV infection. miR-122 inhibitors are currently being used in clinical trials, now entering Phase 3 [39]. miR-33 inhibition raises atheroprotective plasma high density lipoprotein (HDL) cholesterol while lowering very low density lipoprotein (VLDL) cholesterol in non-human primates [38]. This suggests that anti-miR-33 therapies, now also entering human clinical trials, are an effective approach in ameliorating plasma cholesterol profiles in patients.

### 5.3. Vascular Oxidative Stress

Regarding the importance of cellular responses to redox imbalance in vascular disease, certain miRNAs are crucially modulated. Magenta et al. [64] recently reviewed the role of different miRNAs in EC and VSMC oxidative pathophysiology. In ECs responsive to oxidative stress induced by hydrogen peroxide (H_2_O_2_) stimulation, miR-200 family members were upregulated. Its role in redox signalling is likely exerted through Zinc Finger E-Box Binding Homeobox (*ZEB1*) inhibition. NO stimulation also increased miR-200 family miRNAs and inhibited another ZEB splice variant, *ZEB2*. Modulation of these proteins could indicate a role for miR-200 in ROS-induced apoptosis and senescence (ZEB1) and cardiovascular development (ZEB2). *Silent mating type information regulation 2 homolog* (SIRT1) is a longevity-associated enzyme important in cellular metabolism in general, and specifically in EC response to oxidative stress [64]. miR-200a [65], miR-34a [66], miR-92a [67], miR-199a [68], and miR-217 [69] have been shown to affect *SIRT1* function *in vitro*.

Vascular occlusion resulting in ischemia triggers a hypoxic response in affected cells. Under hypoxic conditions, mitochondrial ROS production is increased, generating an oxidative environment. To date, miR-210 is the most prominent miRNA in hypoxia. It is produced in response to hypoxia inducible factor (HIF) transcription factor activation and affects a number of target genes involved in many different cellular pathways [70]. It is suggested that miR-210 deregulation might have a detrimental role in the cellular response to hypoxia-induced oxidative stress, but the mechanisms responsible are not yet entirely clear [64].

Deletion in mice of miR-378/378*, two miRNAs derived from the same hairpin precursor, produces animals protected against diet-induced obesity [71]. It was shown that both miRNAs are involved in energy homeostasis via carnitine *O*-acetyltransferase (CRAT) targeted by miR-378 and mediator subunit complex 13 (MED13) targeted by its passenger strand miR-378*. Mice with miR-378/378* knocked out displayed increased energy expenditure and mitochondrial oxidative capacity in insulin target tissues such as adipose and skeletal muscle tissue. These findings make miR-378/378* interesting drug targets.

Very few studies have until now focused on the role of miRNAs in cardiovascular AGE/RAGE signalling. Togliatto et al. showed in HUVECs that miR-221/222 downregulation is important in AGE- and high glucose mediated cell cycle arrest by downregulation of cyclin-dependent kinase inhibitors 1B and 1C (*CDKN1B* or p27^Kip1^ and *CDKN1C* or p57^Kip2^, resp.) [72]. In colon cancer, miR-155 is regulated in a RAGE-responsive manner [73]. *In vitro* exposure of human monocytes to AGEs induced miR-214 production and subsequent phosphatase and tensin homolog (*PTEN*) downregulation in these cells. By luciferase reporter assay, PTEN was validated as a miR-214 target [74].

## 6. Circulating Inflammatory Mediators and miRNA Involvement

The proinflammatory function of adipokines is an important general mechanism in diabetes-related vascular dysfunction.

### 6.1. Adipokines

These adipose tissue-derived cytokines show a complex interplay with different miRNAs. Investigating miRNAs in adipose tissue from subjects of Indian descent, Meerson et al. found that miR-221 abundance was correlated with obesity. miR expression was negatively regulated by the adipokine leptin, as well as by TNF-*α* [75]. The authors found that miR-221 suppressed the adiponectin receptor 1 (*ADIPOR1*) and the transcription factor v-ets erythroblastosis virus E26 oncogene homolog 1 (*ETS1*) in HEK293 cells. On the mRNA level, this function was not observed in adipose tissue, but at the protein level, both ADIPOR1 and ETS1 were reduced. This reduction could lead to changes in insulin sensitivity and promote obesity-associated inflammation [75].

## 7. Vascular Cell-Specific miRNA Stress Responses

**Figure 3:**
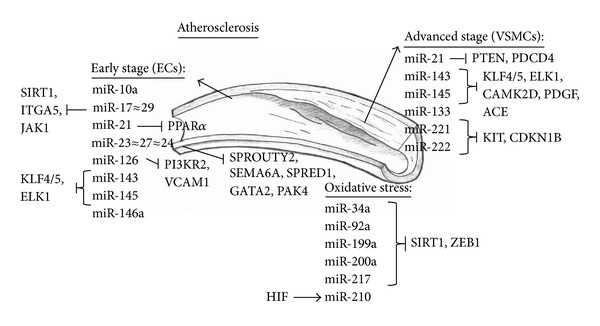
miRNAs involved in vascular pathophysiology. ECs: endothelial cells; VSMCs: vascular smooth muscle cells; SIRT1: silent mating type information regulation 2 homolog; ITGA5: integrin subunit *α*5; JAK1: Janus kinase 1; KLF: Krüppel-like factor; ELK1: ETS domain-containing protein Elk1; PPAR*α*: peroxisome proliferator-activated receptor *α*; SPROUTY2: sprouty protein 2; SEMA6A: semaphorin-6A; SPRED1: sprouty-related, EVH1 domain-containing protein 1; GATA2: GATA-binding protein 2; PAK4: p21 protein-activated kinase 4; PI3KR2: phosphatidylinositide 3-kinase regulatory subunit 2; VCAM1: vascular cell adhesion molecule 1; HIF: hypoxia-inducible factor 1; ZEB1: Zinc Finger E-Box Binding Homeobox 1; PTEN: phosphatase and tensin homolog; PDCD4: programmed cell death 4; CAMK2D: calcium/calmodulin-dependent protein kinase II delta; PDGF: platelet-derived growth factor; ACE: angiotensin-converting enzyme; KIT: v-kit Hardy-Zuckerman 4 feline sarcoma viral oncogene homolog; CDKN1B: cyclin-dependent kinase inhibitor 1B.

Different cell types may respond to, and themselves release, different miRNAs. Certain miRNAs, for example, that are scarce in nucleated cells might have profound actions in platelets [76]. Different cell types might present different miRNA targets, which demands an even more careful approach towards miRNA expression interpretation. We will present the most influential cell types that constitute the vasculature, as well as the miRNAs proven to have a role in their pathophysiology (Figure 3).

### 7.1. Endothelial Cells

miR-17*≈*92, -21, -23*≈*27*≈*24, -126, -143, -145, and -146a have the most extensive record in endothelial cell physiology and pathology [77–82]. Interaction of these miRNAs with ECs affects the cells' angiogenesis, sprouting, and vascular remodelling capabilities via *SIRT1*, integrin subunit *α*5 (*ITGA5*), and Janus kinase 1 (*JAK1*) (miR-17 *≈* 92) [67, 77, 79, 80], Sprouty protein 2 (*SPROUTY2*) and Semaphorin-6A (*SEMA6A*) (miR-23 and -27) [80], and Sprouty-related, EVH1 domain-containing protein 1 (*SPRED1*), phosphatidylinositide 3-kinase regulatory subunit 2 (*PI3KR2*/*p85*/**β**), and vascular cell adhesion molecule 1 (*VCAM1*) (miR-126) [44]. miR-21 inhibits EC inflammation through peroxisome proliferator-activated receptor *α* (*PPAR*α**) [81]. miR-24 mediates EC apoptosis via GATA-binding protein 2 (*GATA2*) and p21 protein-activated kinase 4 (*PAK4) *[82]. Endothelial cell-derived miR-143/145 can repress ETS domain-containing protein Elk1 (*ELK1*), Krüppel-like factor 4 (*KLF4*), and calcium/calmodulin-dependent protein kinase II delta (*CAMK2d*) in VSMCs [77]. Exactly how this kind of communication is established has developed into its own independent small area of research.

EC injury triggers a release of endothelial cell derived microparticles (EMPs) [83]. EMPs have a range of functions in vascular homeostasis, such as coagulation, inflammation, endothelial function, and angiogenesis [83] and are rich in miRNAs, particularly miR-126 [84]. Recent work by Jansen et al. shows that miR-126 is reduced in circulating EMPs of patients with T2DM versus non-diabetic controls and contributes to EMP-mediated regeneration of target cells *in vitro *and *in vivo* [84]. What remains to be investigated is if therapeutic reconstitution of miR-126 containing EMPs in patients with T2DM can reverse the vascular pathology observed in this disease. Other groups have also showed that loss of endothelial miR-126 is part of the T2DM plasma miRNA signature, as mentioned previously [44].

Another component in vascular repair are endothelial progenitor cells (EPCs). Via paracrine routes, these circulating CD34+/CD133+ VEGFR2+ immature hematopoietic cells could orchestrate the reaction of the endothelium to injury [85, 86] and EPC function might explain, in part, the beneficial effects of statins in cardiovascular disease [87]. In circulating EPCs from T2DM patients, miR-21, -27a, -27b, -126, and -130a are downregulated [88]. miR-130a inhibition in EPCs reduces the proliferation, migration, and colony formation of these cells *in vitro*. Runt-related transcription factor 3 (*RUNX3*) is a direct target of miR-130a and reduction of Runx3 protein level rescues EPC proliferation, colony formation, and migration. In these particular cells, these are effects likely to be evoked by miR-130a [88].

Less well-known modulators of endothelial cell behaviour are miR-10a, promoting EC inflammation via inhibition of homeobox A1 (*HOXA1*), mitogen-activated protein 3 kinase 7 (*MAP3 K7*), and *β*-transducin repeat containing E3 ubiquitin protein ligase (*BTRC*), and miR-210, a proangiogenic miRNA inhibiting ephrin-A3 (*EFNA3*) [70, 89]. On the opposite side, miRNAs miR-221 and -222, acting through *v-kit Hardy-Zuckerman 4 feline sarcoma viral oncogene homolog* (KIT)-ligand (*KITLG*) and signal transducer and activator of transcription 5A (*STAT5A*), inhibit angiogenesis [90, 91]. *In vitro *experiments in HUVECs recently identified miR-503 as a regulator of EC cycle progression via the phosphatase cell division cycle 25A (*CDC25A*) and cyclin E1 (*CCNE1*) [92]. In early phases of endothelial dysfunction, miR-146a is primarily involved in the regulation of inflammation, targeting toll-like receptor pathway signals [93]. Interestingly, miR-146a is also involved initial phases of type 1 diabetes in nonobese diabetic mice, acting on NF-*κ*B responsive genes in *β* cells [55].

### 7.2. Vascular Smooth Muscle Cells

Progressing atherosclerotic plaques are characterised by migration of VSMCs towards the lesion [20]. The fibrous cap they create promotes plaque stability. VSMC interplay with the inflammatory environment leads to LDL cholesterol accumulation and ROS generation, as well as VSMC apoptosis, which both aggravate the inflammatory reaction and lead to plaque rupture. VSMC survival, proliferation, migration, and remodelling are governed by miR-143 and -145 via their targets *KLF*4 and 5, *ELK1*, *CAMK2D*, platelet-derived growth factor (*PDGF*), and angiotensin I converting enzyme (*ACE*). miR-21 inhibits *PTEN* and programmed cell death 4 (*PDCD4*) and inhibits the apoptosis regulator B-cell CLL/lymphoma 2 (*BCL2*), thereby promoting VSMC proliferation, a mechanism validated in murine models of vascular disease [94, 95]. miR-221 and miR-222 contribute to VSMC dedifferentiation and proliferation by targeting the tyrosine-protein kinase *KIT*, as well as *CDKN1B *and* CDKN1C* [96, 97]. *In silico* analysis showed that miR-133 could have the potential to block VSMC phenotypic switching through the transcription factor Sp-1 [98].

## 8. lncRNAs and Diabetes

Genome-wide association studies have identified *antisense noncoding RNA in the INK4 locus* (*ANRIL*), a lncRNA discovered in 2007 and associated with neural system tumours [99], as a genetic locus of susceptibility for coronary disease, intracranial aneurysm, and T2DM [100, 101]. Its host loci, cyclin-dependent kinase inhibitors 2A and 2B (*CDKN2A/B*), encode for tumour suppressor genes. This implicates that *ANRIL* might affect cellular senescence and replicative function and thus influences the molecular mechanisms involved in these diseases [102]. In human peripheral blood mononuclear cells (PBMCs) and the monocytic cell line MonoMac, *ANRIL*, guided by its core sequence called the Alu motif, was shown to bind to different proteins contained in the chromatin modifying complexes polycomb repressive complex (PRC) genes. The inhibitory and activation functions affected atherosclerosis-related cell functions such as adhesion, proliferation, and apoptosis, and perhaps surprisingly not *CDKN2A/B* [103].

There are >1100 human *β*-cell lncRNAs. They are an integral component of the *β*-cell differentiation and maturation program. *HI-LNC25*, for example, regulates GLI-similar zinc fincer protein 3 (*GLIS3*) mRNA, an islet transcription factor. Two other lncRNAs, *KCNQ1OT1* and *HI-LNC45*, are up-, respectively, downregulated in human T2DM pancreatic islets. Two lncRNAs map within the established T2DM susceptibility loci prospero homeobox 1 (*PROX1*) and Wolfram syndrome 1 (*WFS1*) [104].

lncRNAs in the rat genome play a role in the response of rat VSMCs to angiotensin II. Associated lncRNAs are *Lnc-Ang26*,* Lnc-Ang383*, *Lnc-Ang58*, *Lnc-Ang219*, *Lnc-Ang202*, *Lnc-Ang249*, and *Lnc-Ang362*. *Lnc-Ang362* is a host gene for miR-221 and miR-222, which means that these miRNAs are cotranscribed with and excised from the lncRNA. miR-221 and miR-222 are miRNAs known to regulate VSMC proliferation [43].

## 9. Summary and Conclusion

The rapid rise of T2DM and its accompanying cardiovascular complications demand new treatment strategies. The widespread possibilities of modulating gene expression using various subtypes of ncRNAs present great opportunities to fight the burden of the associated diseases. However, before initiating treatment of patients by modulating ncRNA, the in-depth mechanisms of their action and regulation need to be completely understood. ncRNA interaction, translation to humans, drug delivery, off-target side effects, and many other challenges still require thorough basic and translational studies. However, utilizing modern technology (e.g., microarrays, RNAseq) has enabled us to discover ncRNA regulation of disease, which makes the identification of novel treatment options in cardiovascular disease more rapid compared to traditional avenues of drug design. This may allow us to get closer to a solution for the rapidly expanding worldwide health problem of T2DM and its related pathologies.
